# Advancing utilization of diverse global carrot (*Daucus carota* L.) germplasm with flowering habit trait ontology

**DOI:** 10.3389/fpls.2024.1342513

**Published:** 2024-04-19

**Authors:** Jenyne Loarca, Michael Liou, Julie C. Dawson, Philipp W. Simon

**Affiliations:** ^1^ Vegetable Crops Research Unit, United States Department of Agriculture, Madison, WI, United States; ^2^ Department of Plant and Agroecosystem Sciences, University of Wisconsin-Madison, Madison, WI, United States; ^3^ Department of Statistics, University of Wisconsin-Madison, Madison, WI, United States

**Keywords:** plant genetic resources, plant breeding, diverse germplasm, biennial crops, flowering habit, annual crops, crop resilience, crop wild relatives

## Abstract

Biennial vegetable crops are challenging to breed due to long breeding cycle times. At the same time, it is important to preserve a strong biennial growth habit, avoiding premature flowering that renders the crop unmarketable. Gene banks carry important genetic variation which may be essential to improve crop resilience, but these collections are underutilized due to lack of characterization for key traits like bolting tendency for biennial vegetable crops. Due to concerns about introducing undesirable traits such as premature flowering into elite germplasm, many accessions may not be considered for other key traits that benefit growers, leaving crops more vulnerable to pests, diseases, and abiotic stresses. In this study, we develop a method for characterizing flowering to identify accessions that are predominantly biennial, which could be incorporated into biennial breeding programs without substantially increasing the risk of annual growth habits. This should increase the use of these accessions if they are also sources of other important traits such as disease resistance. We developed the CarrotOmics flowering habit trait ontology and evaluated flowering habit in the largest (N=695), and most diverse collection of cultivated carrots studied to date. Over 80% of accessions were collected from the Eurasian supercontinent, which includes the primary and secondary centers of carrot diversity. We successfully identified untapped genetic diversity in biennial carrot germplasm (n=197 with 0% plants flowering) and predominantly-biennial germplasm (n=357 with <15% plants flowering). High broad-sense heritability for flowering habit (0.81 < H^2^< 0.93) indicates a strong genetic component of this trait, suggesting that these carrot accessions should be consistently biennial. Breeders can select biennial plants and eliminate annual plants from a predominantly biennial population. The establishment of the predominantly biennial subcategory nearly doubles the availability of germplasm with commercial potential and accounts for 54% of the germplasm collection we evaluated. This subcollection is a useful source of genetic diversity for breeders. This method could also be applied to other biennial vegetable genetic resources and to introduce higher levels of genetic diversity into commercial cultivars, to reduce crop genetic vulnerability. We encourage breeders and researchers of biennial crops to optimize this strategy for their particular crop.

## Introduction

In sexually reproducing crop plants, flowering is essential for breeding and seed production. Flowering phenology has important implications for other agronomic traits, such as plant biomass, disease onset, fruit ripening, seed yield, marketability, and harvest window - as such, there has been great interest in characterizing crop germplasm collections for phenological flowering data, as has been done in lentil (*Lens culinaris* Medik.) (Tullu et al., 2008), turnip and rutabaga (*Brassica napus* L.) (Cruz et al., 2007), safflower (*Carthamus tinctorius* L.) (Elfadl et al., 2010), honeysuckle (*Lonicera caerulea* L.) (Gerbrandt et al., 2017), grape (*Vitis vinifera*) (Wolkovich et al., 2017), blueberry (*Vaccinium* species) (Campa and Ferreira, 2018), cassava (*Manihot escuentla* Crantz) (Silva Souza et al., 2020), olive (*Olea europaea* L.) (Belaj et al., 2020), peach (*Prunus persica* L.) (Atagul et al., 2022), and common bean (*Phaseolus vulgaris* L.) (Basavaraja et al., 2023).

In many commercial vegetable crops, premature initiation of flowering stem (bolting) or flower primordia (early-flowering), has a well-documented severe adverse impact on yield and quality, especially for vegetable crops with both annual and biennial lifeforms. Many plants that provide global human sustenance originated as wild annual plants that have been selected for delayed annual flowering or biennial flowering habit. As such, many commercial vegetables can have annual or biennial lifeforms, with annual lifeforms being adapted to warmer climates and requiring less time in cold exposure to flower, and biennials requiring more cold exposure (and a second season of growth) to induce flowering. Before bolting, these vegetables have edible tender shoots and/or nutritious storage organs. These vegetables have significant culinary and nutritional properties, as well as cultural, social, and economic value. They are encompassed by a few taxonomic families, including *Apiaceae* (root vegetables such as carrot and parsnip, as well as culinary herbs such as leaf parsley, cilantro, fennel, and caraway); *Alliaceae* (culinary vegetables such as onion, shallot, and leek); *Amaranthaceae* (with edible roots such as beet and leafy greens such as chard and spinach); and *Brassicaceae*, a family with many species used as vegetables due to highly diversified edible vegetative storage organs. With the exception of genus *Raphanus* (radish and daikon), most are from the genus *Brassica* and are dispersed across six species, including *B. oleracea*: leaves (cabbage, collard greens, kale), stems (kohlrabi), and buds (Brussels sprouts); *B. rapa*: root (turnip), seeds (field mustard), and leaves (Napa cabbage (Chinese cabbage), bok choi, and rapini); *B. napus*: root (rutabaga); and the culinary spice, mustard seed, from *B. nigra*, *B. juncea*, and *B. carinata*.

Premature bolting in many vegetable crops results in significant economic losses, due to coincidence with root lignification, production of bitter secondary metabolites, and limited yield potential as plant reserves are shuttled toward reproductive rather than vegetative growth. When these plants transition from their vegetative growth phase to their reproductive growth phase, they channel resources to the growth and development of the seed stalk; this in turn causes rapid deterioration of the vegetative tissues, which senesce and become unmarketable (Quiros, 1993). In vegetable crops where the economic product is the vegetative shoot tissue, bolting initiates biochemical changes that cause edible vegetative shoot tissue to become unpalatable due to damage and hardening from senescence in lettuce (Rosental et al., 2021), Chinese cabbage (Yui and Yoshikawa, 1991; Wang et al., 2014; Jiang et al., 2023), celery (Quiros et al., 1987; Quiros, 1993), and spinach (Ribera et al., 2020), as well as to secretion of latex and bitter secondary metabolites in lettuce (Ciriaci et al., 2013) and spinach (Abe et al., 2014). In vegetable crops where the fleshy storage root is the economic product, bolting gives way to root lignification, preventing tap root thickening (Villeneuve, 2020), and rendering an inedible, woody, unmarketable product, thus causing serious economic losses to growers of carrot (Dowker and Jackson, 1975; Prohens and Nuez, 2008; Simon and Grzebelus, 2020), table beet (Holland and Dowker, 1969; Dowker et al., 1971; Goldman, 2004), onion (Khokhar et al., 2007; Hyun et al., 2009; Baldwin et al., 2014; Havey, 2018), and turnip (Nishioka et al., 2005).

Breeding for bolting resistance or toward delayed flowering has long been recognized as a solution to premature flowering in vegetables crops with annual and biennial lifeforms, and has been cited as a priority breeding objective in all of the aforementioned vegetables, as well as celeriac (Bruznican et al., 2020), semi-tropical beet (McGrath and Panella, 2018), and chard (Colley, 2017). Evaluation of flowering time in diverse germplasm collections of vegetable crops with annual and biennial lifeforms has resulted in the identification of accessions with delayed bolting or non-bolting genotypes in carrot (Tabor et al., 2016), arugula (Morales et al., 2006), coriander (Bashtanova and Flowers, 2011), spinach (Chitwood et al., 2016), lettuce (Jang et al., 2019; Lebeda et al., 2019), and caraway (Von Maydell et al., 2024), suggesting that this is a viable strategy to successfully identify germplasm with commercial potential for use in breeding programs. The longstanding recognition that biennial flowering habit has a demonstrated strong genetic underpinning means that breeding delayed-bolting or bolting-resistant cultivars is a powerful, economical, and achievable strategy to improve this critical trait.

Despite these success stories and the knowledge that landrace varieties harbor great genetic potential for beneficial traits that promote crop resilience, commercial crop breeders are reluctant to utilize genebank germplasm due to linkage drag, or the unintended coinheritance of undesirable alleles alongside a trait of interest (Zamir, 2001; Bohra et al., 2022). The perception is realistic that genebank accessions could be difficult to work with due to challenges related to desirable traits being in linkage with poor agronomic performance traits such as premature flowering; thus, lack of phenological flowering data remains a significant deterrent to utilization of plant genetic resources in many crop improvement programs (Dempewolf et al., 2017; Zamir, 2001; Bohra et al., 2022). This is an especially daunting prospect in breeding biennial crops, given that genetic gain is limited by cycle time, and biennial crops achieve at most one cycle per year. We are thus at risk of not utilizing important genetic diversity for other key traits that growers need, rendering crops more vulnerable to diseases, pests, and other environmental stresses. The impetus to characterize germplasm based on critical phenological flowering traits is a logical starting point to advance utilization and prioritization of biennial crop genetic resources.

Carrot (*Daucus carota* ssp. *sativus*) is a vegetable crop with a relatively recent domestication history (~900 years ago, to date), known for being a significant source of dietary fiber and provitamin A carotenoids (Simon et al., 2019). Major primary domestication syndrome traits in root crops like carrot include biennial growth habit, the ability to form a fleshy storage root from secondary growth, and reduced lateral root branching (Macko-Podgórni et al., 2017; Ellison, 2019). Recent molecular studies have confirmed that domesticated carrots were derived from wild populations of Central Asian *D. carota* ssp*. carota*, also known as Queen Anne’s Lace (Iorizzo et al., 2013). The emergence of biennial carrot plants from annual types was a consequence of human-mediated selection for maximal vegetative growth prior to reproduction (Goldman, 2004). Selection under the process of domestication after carrots arrived in Europe modified the life cycle of carrots from annuals to biennials, thereby ensuring a full summer season of vegetative growth without floral initiation. Whether consciously or unconsciously, European farmers and breeders leveraged this natural genetic adaptation, using carrot’s large vegetative reserves as sustenance in colder climates. Selection for a larger taproot and short growing season in colder climates necessitated a biennial lifecycle for carrot to achieve maximum vegetative growth without reduction of consumer quality. Consequently, the biennial carrot was derived from its wild annual progenitor by prioritizing vegetative traits and eliminating annual reproductive growth. Biennial growth habit is critical for non-woody, succulent storage root development that can be used as a food crop, and was undoubtedly one of the first selected traits in the lineage that become domesticated carrot (Macko-Podgórni et al., 2017; Ellison et al., 2018; Ellison, 2019). As such, biennial growth habit is biologically linked to fleshy root storage, the hallmark domestication trait in carrots. However, as a group, annual cultivated carrots developed for subtropical and semi-arid regions are at least as fleshy as biennial carrots.

Broad variation in bolting and flowering initiation reflects the ecological adaptations of plants to their local climatic conditions (Lebeda et al., 2019). In carrot, vernalization time requirement is genotype-dependent (Wohlfeiler et al., 2021), and variation for time requirement has been reported within annuals and biennials, with annuals needing shorter periods of cold exposure (5°C or 41°F from 5 to 30 days) and biennial cultivars requiring longer period of cold exposure (11–12 weeks) to initiate floral stem elongation and flower morphogenesis (Linke et al., 2019; Wohlfeiler et al., 2022). ‘Annual’ refers to plants that flower without a vernalization requirement and in the first growing season, or first season in which the seed is planted. In nature, vernalization is a natural genetic adaptation to environments in which it is advantageous to delay flowering in favor of a period of vegetative growth. Energy reserves that accumulate in root tissues of biennial root crops during the first season of growth fuel reproductive structure development during the second season of growth. Without vernalization, an obligate biennial carrot may never flower.

The genetic control of carrot flowering is under extensive study. A region on the distal arm of chromosome 2 has been implicated by several independent studies as a likely target region during the course of carrot domestication. In this region, two overlapping selective sweeps (Grzebelus et al., 2014; Ellison et al., 2018) are in close proximity to the vernalization gene *Vrn1* (Alessandro et al., 2013) and a candidate domestication gene (DcAHLc1) involved in root tissue thickening (Macko-Podgórni et al., 2017). Furthermore, the candidate domestication syndrome gene (DcAHLc1) systematically differentiates wild and cultivated accessions, and it is hypothesized that this gene is involved in the development of the carrot storage root, as the localization of the gene overlapped with one of the QTL for root thickening. In the most extensive investigation of carrot flowering-time regulation genes, 45 unigenes were identified (Ou et al., 2017), including three putative FLOWERING LOCUS (FLC) genes, which are known to delay or repress flowering in *Arabidopsis* (Michaels and Amasino, 2000). These putative FLC genes were also differentially expressed between wild carrots and domesticated carrots (Ou et al., 2017). Taken together, these studies confirm biennial growth habit is a genetically controlled trait that was a primary target during domestication (Alessandro et al., 2013; Ellison, 2019). More recently, it was found that post-vernalization day length does not influence carrot flowering (Wohlfeiler et al., 2022).

Over 13,400 *Daucus* accessions are conserved globally by 62 institutions (Allender, 2019), yet essential phenological data, such as flowering habit, is not available for many accessions. Phenological flowering data is critical to selecting locally adapted and commercially relevant germplasm to screen for a breeding program. For example, in semi-arid and subtropical climates, temperatures rarely achieve the sustained lows required for vernalization, and large-volume refrigerated coolers for vernalization are unavailable (Simon & Grzebelus, 2020). As such, carrots cultivated in this region require late-flowering annual habit; some carrot plants are used to produce the root crop, and some generate the seed stock later in the same season. Biennial plants are unsuited to globally warm regions as they cannot contribute to the seed crop in subtropical/semi-arid markets. In contrast, cultivated carrots grown commercially in temperate climates, such as Europe, North America, and Australia, are of obligate-biennial stock (Goldman, 2004). Flowering at any time in the first season of growth (annual-flowering habit, whether early-flowering or late-flowering) is problematic and intolerable in temperate climates of commercial carrot root production (Rubatzky et al., 1999; Prohens & Nuez, 2008). As with other biennial vegetable crops, the transition from the vegetative to reproductive phase in carrot coincides with rapid lignification of the xylem, even before the floral stalk/bolting stem elongates, rendering the roots fibrous and inedible, and resulting in complete loss of consumer quality and commercial value (Peterson, 1986; Amasino, 2005; Alessandro & Galmarini, 2007; Ou et al., 2017; Linke et al., 2019; Simon et al., 2019). As such, biennial carrots are required in commercial carrot root production in temperate climates, as annual flowering habit results in complete loss of commercial root crop value and significant economic loss to the grower. For this reason, breeders of temperate carrot routinely select against annual flowering habit (Goldman, 2004).

Few global cultivated carrot germplasm collections have been evaluated for flowering habit or bolting tendency. High broad sense heritability was estimated for bolting tendency among 48 open-pollinated carrot varieties of European and Asiatic origin, studied in India (Manikanta et al., 2018), suggesting genetic potential for improvement. In an evaluation of a carrot germplasm collection (101 accessions) in China, purple rooted accessions demonstrated 48.4% premature bolting tendency, compared with 2.7% - 7% in orange rooted accessions (Bao et al., 2010). This likely reflects breeding efforts toward biennial flowering habit in orange-fleshed roots rather than true genetic linkage of anthocyanin with annual flowering habit. An assessment of 140 U.S. commercial carrot cultivars noted negligible amounts of bolting in this panel (Luby et al., 2016). Similarly, few wild carrot germplasm collections have been evaluated for flowering habit or bolting tendency. A recent study of 14 wild Nordic carrots (*Daucus carota* subsp. *carota*) found that sowing time had a strong influence on flowering time, with earlier sowings resulting in increased annual behavior (Solberg and Yndgaard, 2015). A similar study of 10 wild carrot accessions found high inter- and intra-accession variation for flowering time in multi-environmental trials, with percent-flowering having a significant location effect and an insignificant genotype effect (Geoffriau et al., 2019), suggesting low genetic diversity for flowering in this population, high environmental influence, or both. The range of results reflects the variation in flowering habit across carrot germplasm collections.

Whereas premature bolting is an irredeemable trait in commercial crop production, it is possible and necessary to disentangle the undesirable early-bolting accessions from desirable late-flowering annuals and biennials to optimize use of genetic resources. Given the critical role of plant genetic resources in crop resilience, it is important to have the ability to use a wider range of genetic resources for resistance to emerging diseases, pests and environmental stresses. There may be many genetic resources suitable to various global production environments and market needs which are not being used due to the perception that they will bring in undesirable flowering habits. As such, characterization of flowering phenology has great potential to increase engagement with plant genetic resources, increase levels of genetic diversity in commercial crop cultivars, and reduce genetic vulnerability to shifts in production conditions for these nutritionally, economically, culinarily, and culturally important biennial vegetables.

## Materials and method

### Population under study

*Daucus* accessions (N=1381) are maintained through the U.S. National Plant Germplasm System (NPGS) at the North Central Regional Plant Introduction Station (NCRPIS) in Ames, IA, with information on the accessions (also known as genotypes or plant introductions) in the Germplasm Resources Information Network (GRIN) database of the NPGS (GRIN-Global, 2023). Each carrot accession is a genetically unique, heterogeneous, heterozygous population. Accessions were selected from the GRIN system for our diversity panel if passport information suggested the presence of domestication traits (N=695). These cultivated carrots represent global carrot germplasm, collected over multiple plant exploration trips between 1947 and 2015 from 60 countries, with over 80% of accessions originating from the Eurasian supercontinent: 53% from Asia, 34% from Europe and the Caucasus, and 13% (in descending order) collected from the Americas, Africa, Australia, and New Zealand. This collection includes 148 total accessions from the primary center of diversity in central Asia (modern-day Afghanistan and surrounding countries) and secondary center of diversity in western Asia (modern-day Turkey) (Vavilov, 1951; Banga, 1957). This collection includes landraces and heirloom cultivars with annual, biennial, or mixed flowering habits. Although biennial flowering habit is a known domestication trait in carrot, reliable accession flowering habit data was not available prior to this study. GRIN-Global maintains flowering habit data for each accession (‘lifecycle’), but this data is not reliable due to being recorded in many different environments and on variable numbers of plants. As such, this data was not a criterion for identifying domesticated germplasm in this evaluation. This study is the first and largest (N = 695 accessions) multi-year field evaluation of flowering habit in a diverse carrot germplasm that includes landraces. The carrot accessions in this study are maintained by the United States Department of Agriculture National Plant Germplasm System (USDA-NPGS). All or parts of this global USDA germplasm collection have previously been evaluated in studies on canopy vigor (Loarca et al., 2024b), core collection curation (Corak et al., 2019), demographic history of carrot domestication and breeding (Coe et al., 2023), genetic structure, phyologeny, and carotenoid presence (Ellison et al., 2018), taproot shape (Brainard et al., 2021), plant growth traits (Acosta-Motos et al., 2021), antioxidant capacity (Pérez et al., 2023), resistance to the necrophytic fungal pathogen Alternaria dauci (Tas, 2016), and several studies on seed germination under abiotic stress (Bolton et al., 2019; Bolton and Simon 2019; Simon, 2019; Simon et al., 2021).

#### Experimental design

In 2016-2018, one plot of appx. 50 seeds from each accession (N=695) were hand-planted in each of two blocks of a randomized complete block design (RCBD) at the Hancock Agricultural Research Station (ARS), located in the central sands region of Wisconsin. Bed preparation and planting methods are described in detail in the companion paper of the present study (Loarca et al., 2024a). In each year, we collected flowering data on 679 - 695 accessions. With data over multiple years, we have characterized flowering habit on 668 accessions.

#### Trait phenotyping

**Figure 1:**
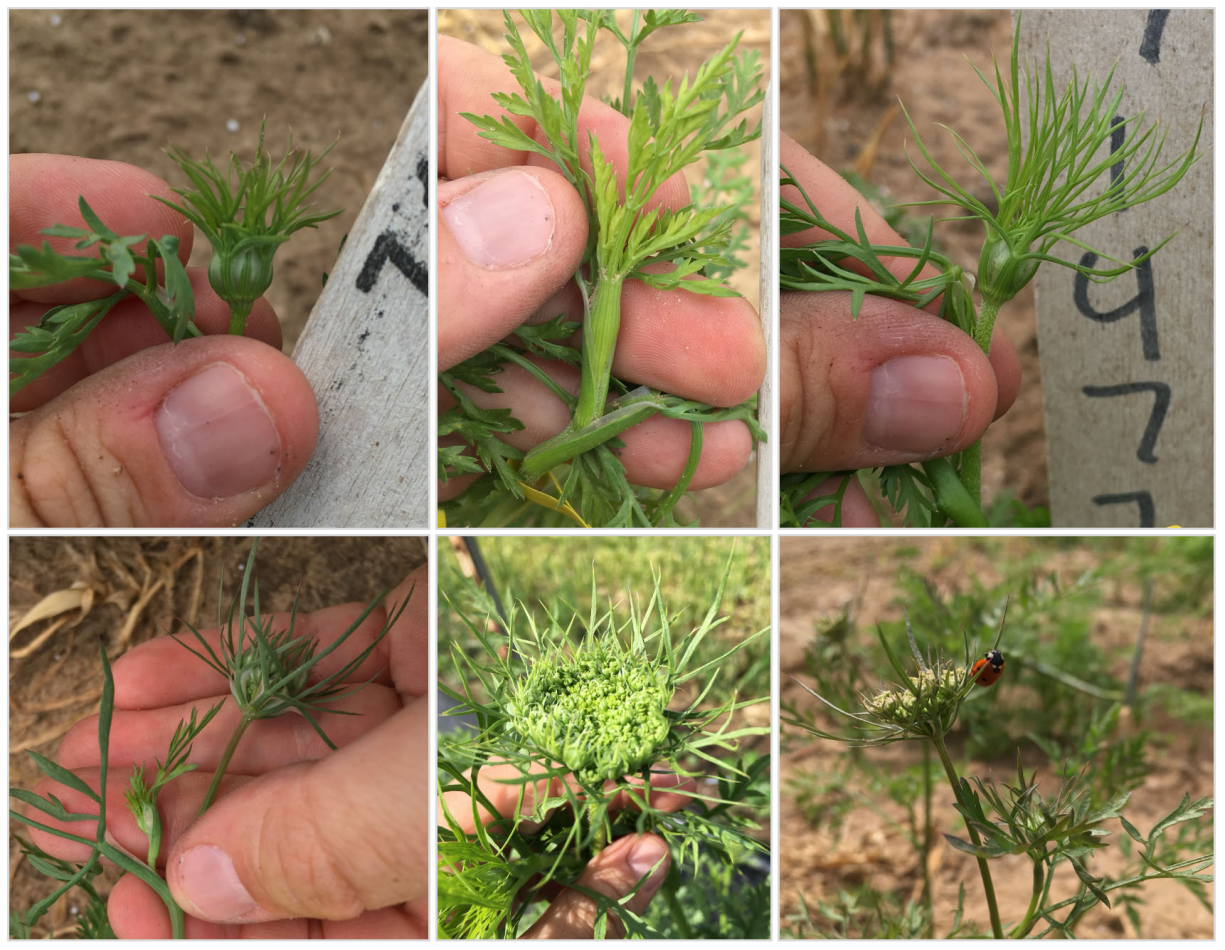
Various expressions of carrot flower primordia at 60 DAS.

Shoot-growth phenotyping methodology is provided in greater detail in the companion paper of the present paper (Loarca et al., 2024a). Flowering habit is a trait that can be assigned to a single plant. Because carrot growth, including the initiation of flowering, can vary widely in carrot, and gene bank accessions are often highly heterozygous, plants from the same accession may express different flowering habits. As such, we phenotyped flowering on a plot-level basis by estimating the percentage of plants showing signs of flowering (**Figure 1**). We scored plots on a 5-point scale (0%, 25%, 50%, 75%, 100%) based on the percentage of plants within each plot with signs of flowering visible to the naked eye, such as stem length greater than 8mm (Villeneuve, 2020) and/or presence of flower primordia, which vary morphologically by genotype and maturity and will require some practice and training to visually identify (**Figure 1**).

Given the undesirability of annual plants for biennial breeding programs, there has been historically no systemic evaluation of flowering habit before end of season (100 DAS in our study). However, it is known that among annual flowering carrots, wild germplasm tends to flower much earlier than cultivated annual germplasm (Simon & Grzebelus, 2020). For this reason, we also scored flowering at 60 DAS, which was the earliest time point at which we observed signs of flowering (**Figure 1**) in 5% - 10% of plots in two of our three studies, and also at 100 DAS (harvest day or end of season). Phenotypic data are stored at the CarrotOmics database (www.carrotomics.org/) (Rolling et al., 2022).

### Data management

We used RStudio Version 2023.6.1.524 (Posit team, 2023) and R Version 4.3.1 (R Core Team, 2023) to perform all statistical analyses. Rosner’s Test in the EnvStats identified multiple simultaneous potential outliers for each trait in each year (Millard, 2013).Various utility packages were crucial to our analysis, such as ggthemes (Arnold, 2021), beepr (Bååth, 2018), flextable (Gohel and Skintzos, 2023), and the tidyverse suite of packages (Wickham et al., 2019).

### Two-way analysis of variance & broad-sense heritability estimation

F-tests of significance were performed to identify significant sources of variation for each trait in each year using fixed effects models in a two-way Analysis of Variance (ANOVA) with Type III sums of squares with the *car* package (Fox et al., 2012/). For each year, a fixed effects model was structured to calculate the proportion of variance in each trait (percentage of flowering plants in plot at 60 DAS or 100 DAS) attributable to genotype and block: *T_ik_* = *u* + *g_i_* + *b_k_* + *e_ik_*, where T_ik_ = phenotype measured on the trait of interest, *u* = intercept, g_i_ = genotype, b_k_ = block, and e_ik_ = error with e_ik_ ~ i.i.d. N(0, σ^2^).

The multi-year fixed effects model includes accessions with trait data across all years and accounts for variation across years: $T_{ijk}=u+g_{i}+y_{j}+{\left(gy\right)}_{ij}+\;b_{k\left(j\right)}+e_{ijk}$ , where T= phenotype of the trait of interest, g_i_ = genotype, y_j_ = year, (gy)_ij_ = genotype*year interaction, b_k(j)_ = block within year, and e_ijk_ = error with each component assumed to follow a respective, independent normal distribution [e_ijk_~ i.i.d. N(0, σ^2^)]. Due to unbalanced data from abnormal weather events (destructive hail), we ran two multi-year analyses: one that included the 2017 flowering data and one that excluded the 2017 flowering data.

Variance components (V) for each trait were estimated using within-year (single-year) and across-years (multi-year) random effects models with the lme4 package (Bates et al., 2015). These used the same model as above with all effects random. Broad-sense heritability (H2) for each trait, within years (single-year model) and across years (multi-year model), was estimated from variance components, including genotypic variance (Vg) and phenotypic variance (Vp). Single-year broad-sense heritability (for each year 2016-2018) was calculated for each trait:


$$H^{2}\;=\;\frac{V_{g}}{V_{p}}=\;\frac{V_{g}}{V_{g}+\;\frac{V_{error}}{\#reps}}$$

Multi-year broad-sense heritability was estimated for each trait:


$$H^{2}\;=\;\frac{V_{g}}{V_{p}}=\;\frac{V_{g}}{V_{g}+\;\frac{V_{gy}}{\#\;years}+\frac{V_{error}}{\#\;years\;*\;\#reps}}$$

#### Mixed models and estimated marginal means

Accessions were categorized into flowering habit based on the estimated marginal means of their percentage flowering at 60 DAS and 100 DAS. We used the same model terms above in a mixed model using the *lme4* package (Bates et al., 2015), with genotype as fixed effect, to extract estimated marginal means for flowering percentage for each accession within and across years with the *emmeans* package (Lenth, 2023). Estimated marginal means on flowering-percentage, within and between years, were used to assign accessions to flowering habit categories based on the 2016 & 2018 data sets.

### Flowering habit ontology

**Figure 2:**
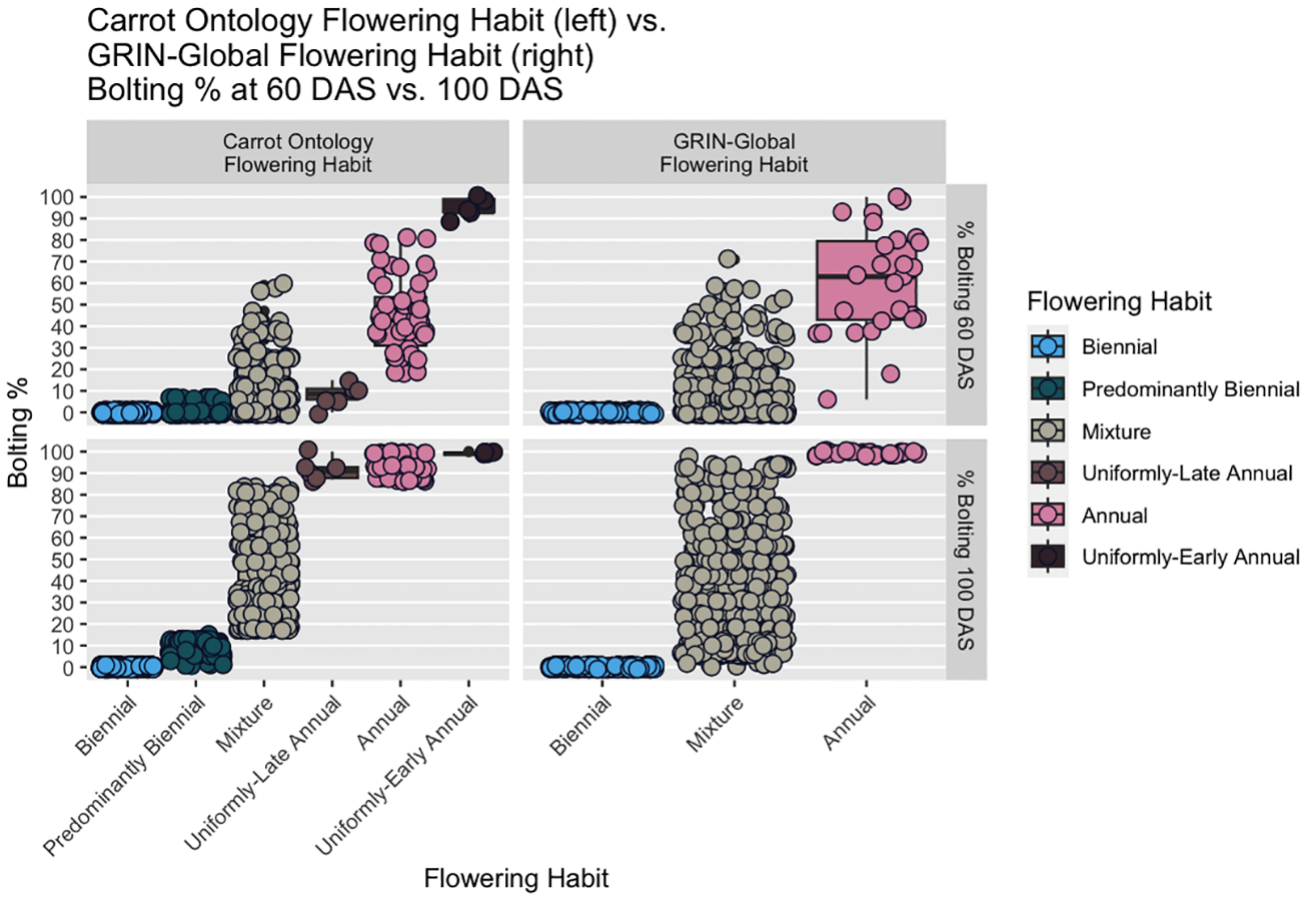
Boxplots of proposed CarrotOmics flowering habit trait ontology (left) vs. 2023 GRIN-Global flowering traits (right) compared with at 60 DAS and 100 DAS. In the new CarrotOmics trait ontology (left), uniformly-early annuals are defined as plots with >85% flowering at 60 DAS and 100 DAS; uniformly-late annuals are plots with ≤15% flowering at 60 DAS and >85% flowering at 100 DAS; and the remaining non-uniformly flowering annuals are plots with <15% < % flowering at 60 DAS < 85% *and* 85% ≤ flowering at 100 DAS). Mixtures (annual and biennial plants) are characterized as 15% < % flowering at 60 DAS < 85% *and* 15% < % flowering at 100 DAS < 85%. In the GRIN-Global panels (right), annual is defined as 100% flowering at 100 DAS.

As of 2023, GRIN-Global describes three categories for carrot flowering habit (or ‘life form’ in the GRIN system): annual, biennial, or a mixture (of annual and biennial plants), however, it was not clear what criteria or thresholds were used to categorize accessions in to one of the three flowering categories. In absence of this information, we created a theoretical construct (‘2023 GRIN-Global Flowering Habit’ in **Figure 2**, right panels) with these three traditional flowering habit categories: annuals had 100% flowering at 100 DAS, biennials had 0% flowering at 100 DAS, and mixtures had between 1% and 99% flowering at 100 DAS.

Starting with these assumptions, we made minor threshold adjustments for the CarrotOmics Flowering Habit Ontology presented in this paper. Given that annual flowering habit is intolerable in temperate regions of carrot root production, we maintained the 0% flowering at 100 DAS threshold for the biennial category. We characterized annual-flowering plants as plots where the vast majority (85% - 100%) of plants were flowering at 100 DAS – despite this slight allowance for non-flowering plants, these are not considered mixed populations. Mixtures had between 1% and 85% of plants flowering at 100 DAS (**Figure 2**, right panels).

**Figure 3:**
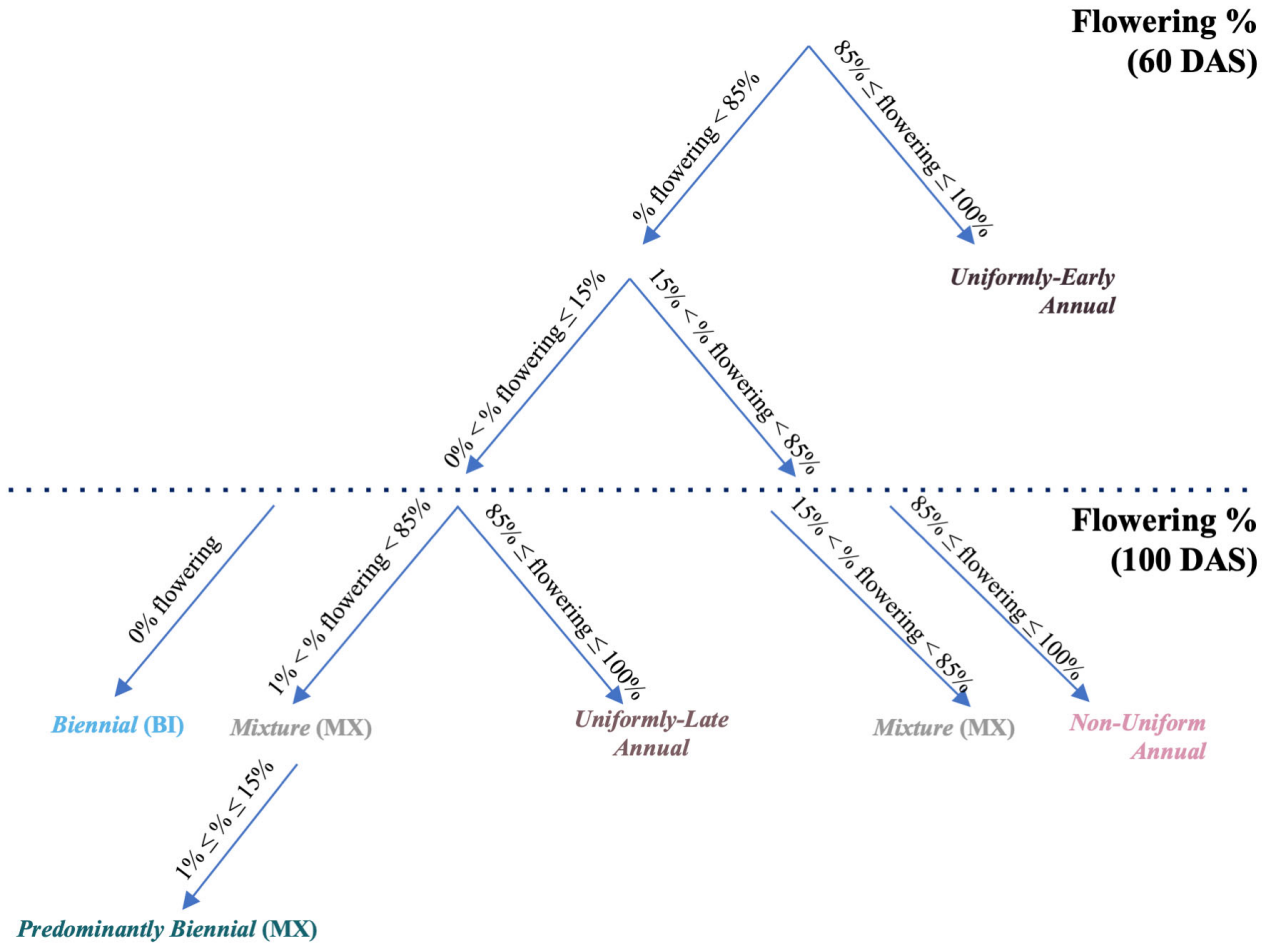
CarrotOmics flowering habit trait ontology flow chart. A visual representation of the flowering habit trait ontology presented in **Table 1**. Assignment of accessions to flowering habit categories is based on their estimated marginal means.

Using this three-category ontology as a baseline, we then subdivide it into different types of annuals and mixtures. We added an additional time-point (60 DAS) for evaluating flowering habit to capture precocity and uniformity of flowering among annuals (**Figure 2**, left panels). We designated annual accessions with 85% - 100% at 60 DAS as *uniformly-early annuals*. Similarly, we designated *uniformly-late annuals* as those with uniform-flowering by the end of the season, with no or low flowering at 60 DAS (≤15%) *and* high amounts of flowering (85% - 100%) at 100 DAS. The remaining annual accessions flower non-uniformly and sporadically between 60 DAS and 100 DAS – these accessions may contain various proportions of uniformly-early and uniformly-late plants, all of which ultimately flower (85% - 100%) by end of season (100 DAS). As described above, mixtures have both biennial and annual plants (1% - 85% flowering at 60 DAS *and* 100 DAS). Recognizing the need for biennial germplasm for breeding and root production in temperate climates, we partitioned some low-flowering mixtures into a predominantly biennial subcategory, which we characterize as having between 1% and 15% flowering at 60 DAS and 100 DAS (**Figure 2**, left panels). This flowering trait ontology (**Table 1** and **Figure 3**) is stored in the CarrotOmics database (www.carrotomics.org/) (Rolling et al., 2022).

**Table 1:** CarrotOmics flowering habit trait ontology developed in this paper, compared with current trait descriptions in GRIN-Global. This paper elaborates on traits that were previously recognized as important in CarrotOmics and provides standard methodologies that carrot researchers can follow, enabling collaboration across programs.

CarrotOmics Trait Ontology for Flowering Habit	2023 GRIN-Global Flowering Trait Descriptors
**Percent flowering (60, 100)**	Percent bolt 1st Year
Percentage of flowering plants within a plot in the first planting season (AKA “first year”) Measured directly from field plots on a 5-point scale (0%-100% in increments of 25%).	Percent bolt in the 1st Year
**Annual flowering habit (60, 100)**	**life cycle. AN=Annual**.
During the first growth season, estimated greater than 85% of plants flowering within the plot.	Annual flowering habit. Plant will flower without a vernalization requirement
** i. Uniformly-early annuals (60)**	
Greater than 85% of plants within plot with signs of flowering at mid-season.	Early flowering field
** ii. Uniformly-late annuals (60, 100)**	**-**
Fewer than 15% of plants within plot with signs of flowering at mid-season and greater than 85% of plants within plot flowering end-of-season. Note: Potentially useful commercial germplasm for subtropical markets.	**-**
** iii. Annuals (non-uniform flowering) (60, 100)**	**-**
Greater than 15% flowering plants within the plot at mid-season and greater than 85% flowering plants within the plot at end-of-season. Note: This is not considered a mixture.	**-**
**Biennial flowering habit (60, 100)**	life cycle. BI = Biennial
0% plants within plot flowering during the first growth season. Roots require vernalization to induce flowering in the second growth season. Note: Potentially useful commercial germplasm for temperature markets.	-
**Mixed flowering habit population (mixture) (60, 100)**	**life cycle. MX = Mixed**
Both annual (uniformly-early and uniformly-late) and biennial plants in various proportions (1% ≤ % flowering< 85%) in the first season of growth. Annuals flower in the first growth season, while biennials’ roots require vernalization in order to flower in the second season of growth.	Mixed population of annual and biennial plants
** Predominantly biennial (60, 100)**	**-**
Type of mixture. Greater than 0% and less than 15% flowering plants in the plot at end of season. Annuals flower in the first growth season, while biennials’ roots require vernalization in order to flower in the second season of growth. Note: Potentially useful commercial germplasm for temperate markets.	**-**

Data collection times may vary by location, cultivar, market type, and length of growing season. Refer to **Figure 3** for logical flowchart of flowering habit ontology.

Data Collection Time (DAS).

“-” indicates no trait descriptor available.

### Trait correlations

Grouped by flowering habit, we evaluated correlations between vegetative growth traits, including seed viability, seed weight, stand count, and canopy height. Pearson correlations were calculated and smoothed trend lines were visualized in a correlation matrix using *GGally*, along with boxplots and scatterplots.

## Results

### Summary statistics of the collection using GRIN-Global flowering habit

The GRIN-Global three-category model characterized biennials (29.5%; n=197) and annuals (9.1%; n=61), with mixtures (61.4%; n=410) representing the vast majority of the collection (**Table 2A**). By 100 DAS, 70.5% of the collection expressed some proportion of flowering. These mixtures are not well-characterized except that accessions in this category contain both annuals and biennials in various proportions.

**Table 2A:** Summary statistics based on 2023 GRIN-Global flowering habit categories (biennial, annual, mixture) at 60 DAS and 100 DAS for all accessions planted 2016-2018.

Flowering Habit	2016		2017		2018		2016 & 2018^i^	
	N=679	%	N=695	%	N=681	%	N=668	%
Biennial	246	36.23	477	68.63	297	43.61	197	29.49
Mixture	373	54.93	172	24.75	307	45.08	410	61.5
Annual	60	8.84	46	6.62	77	11.31	61	9.1

N represents the raw number of accessions in each flowering habit category; percentage (%) indicates the proportion of accessions in the germplasm collection in each flowering habit category. These categories define biennial as 0% flowering, annual as 100% flowering, and mixed as between 1%-99% flowering.

### Summary statistics of the collection using CarrotOmics flowering habit ontology

**Figure 4:**
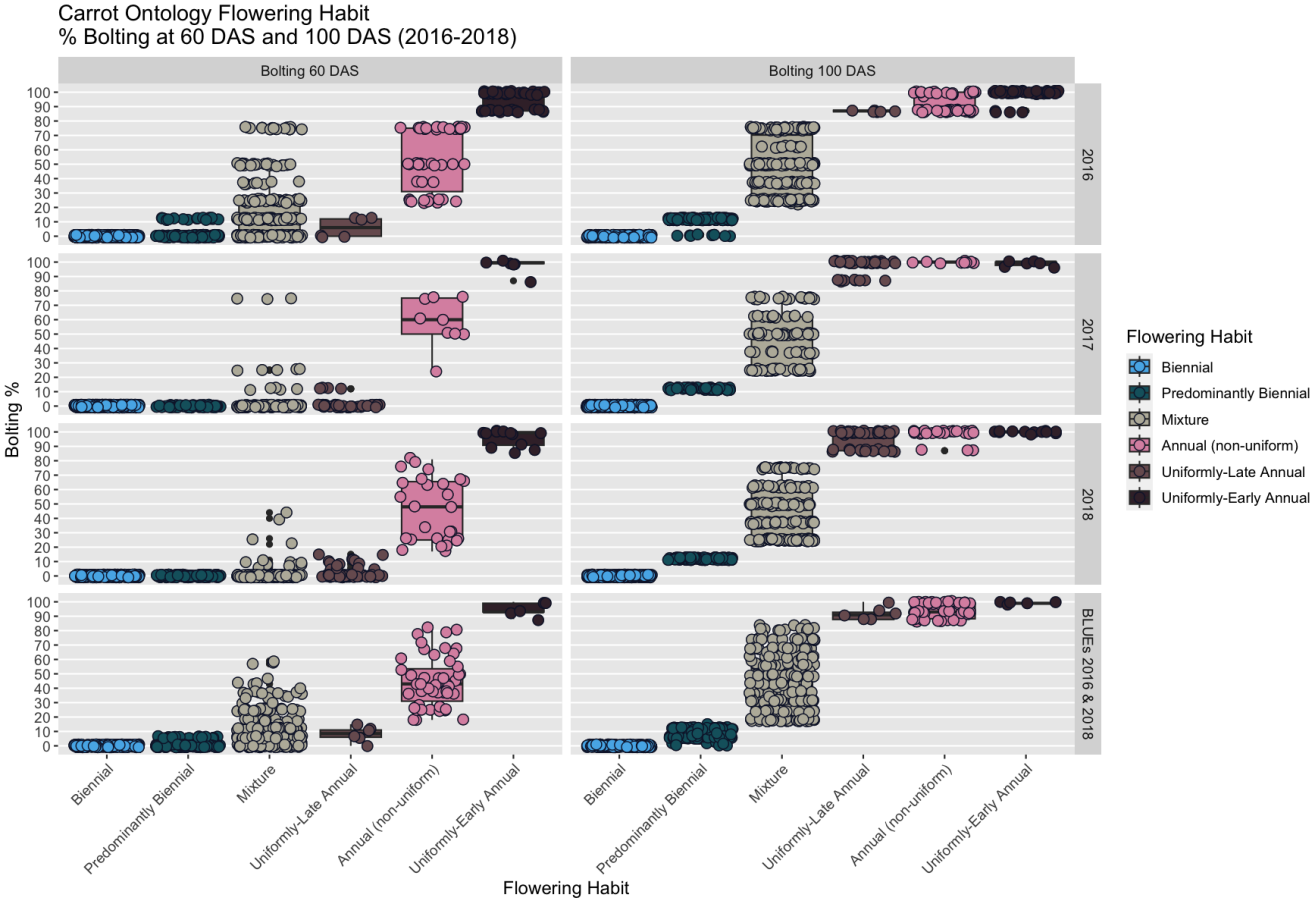
Boxplots of flowering percentage among CarrotOmics flowering habit ontology traits in each year (2016-2018) and averaged across years (2016 & 2018).

Summary statistics are presented using our proposed new ontology to characterize the collection at both the 60 DAS and 100 DAS data (**Table 2B**). Flowering at 60 DAS was the earliest time point at which we observed signs of flowering (**Figure 1**) in 4%-10% of plots in two of our three studies. Flowering thresholds and subcategories did not change the number of biennials identified (n=197), but did increase the number of annuals (10% of accessions; n=61) with fewer than 2% being uniformly-early annuals (n=5) and uniformly-late annuals (n=6). In every year of our evaluation, uniformly-early annuals are a distinct group from uniformly-late annuals at 60 DAS, and both are distinct from the non-uniform flowering annuals (n=50) (**Figure 4**). These remaining annuals flowered non-uniformly with 15% - 85% flowering at 60 DAS *and* 100 DAS.

**Table 2B:** Summary statistics based on CarrotOmics flowering habit trait ontology.

Flowering Habit	2016		2017		2018		2016 & 2018	
	N	%	N	%	N	%	N	%
**Biennial**	**246**	**36.23**	**477**	**68.63**	**297**	**43.61**	**197**	**29.49**
**Mixture**	**332**	**48.90**	**161**	**23.17**	**288**	**42.29**	**410**	**61.38**
Predominantly biennial 0%< % flowering plants ≤ 15%	114	48.90	54	7.77	111	16.30	160	23.95
All other mixtures	218	32.11	107	15.40	177	25.99	250	37.43
**Annual**	**101**	**14.87**	**57**	**8.19**	**96**	**14.09**	**61**	**9.14**
Annual (non-uniform)	47	6.92	9	1.29	27	3.96	50	7.49
Uniformly-early annual	48	7.07	6	0.86	12	1.76	5	0.75
Uniformly-late annual	6	0.88	42	6.04	57	8.37	6	0.90
% Entries Flowering								
Flowering 60 DAS	256	37.70	32	4.60	67	9.84	247	36.98
Flowering 100 DAS	433	63.77	218	31.37	384	56.39	471	70.51

Flowering data for annual, biennial, mixed, and subcategories at 60 DAS and 100 DAS for all accessions planted in 2016-2018. N=number of accessions in flowering habit category; % = percentage of accessions in collection in each flowering habit category. Bold values describe the main three flowering categories (biennial, mixture, and annual), while plain text values (indented) beneath describe the subcategories of each main category.

**Figure 5:**
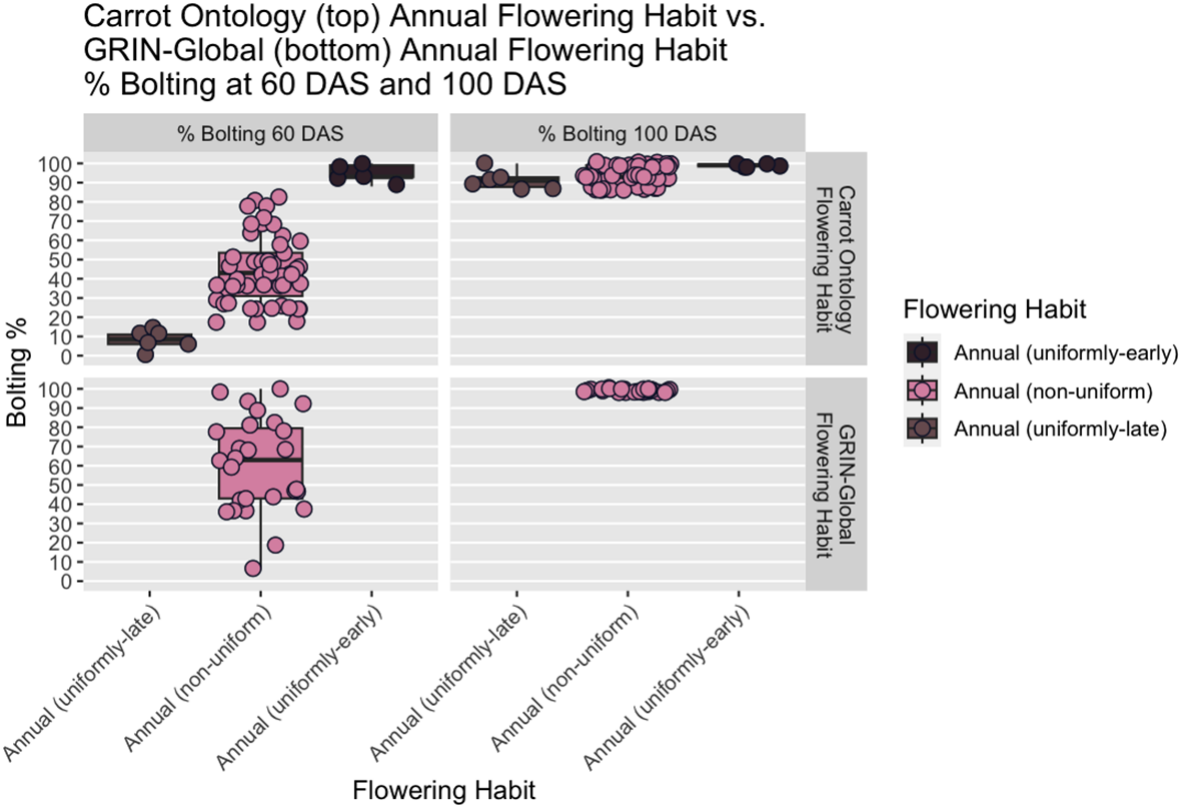
Boxplots showing distinction in flowering percentage among annual-flowering accessions based on proposed CarrotOmics flowering habit trait ontology (top panels). The distinction among uniformly-early annuals, uniformly-late annuals, and non-uniformly-flowering annuals is clear at 60 DAS (left panels) and is lost at 100 DAS (right panel). Flowering percentage among annual accessions using GRIN-Global classification (bottom panels), show that there is no differentiation among accessions at the 100 DAS time point which is used for the classification.

While flowering is typically measured at 100 DAS, the addition of the 60 DAS time-point enabled differentiation between uniformly-early annuals and uniformly-late annuals, a distinction that would otherwise be lost by 100 DAS, as indicated in every year of our study (**Figure 5**). The predominantly biennial subcategory accounts for 24% of accessions (n=160) in the germplasm collection. Flowering percentage among low-flowering mixtures is indiscernible from predominantly biennial populations at 60 DAS, but distinct at 100 DAS. After partitioning the predominantly biennial population, there are 250 mixed-flowering accessions, which have between 15% and 85% annual plants at 100 DAS.

### Analysis of variance & broad-sense heritability

ANOVA results indicate that genotype was a highly significant factor influencing flowering percentage at 60 DAS (**Table 3A**), with broad-sense heritability (H^2^) estimates also consistently very high in each year (**Table 2A**: 0.87< H^2^< 0.93). The block effect was not significant in 2016 or 2018 significant at the p<0.05 level and in 2017. Similarly, F-tests of significance at 100 DAS flowering had a highly significant genotype effect and high broad-sense heritability (**Table 3B**: 0.81< H^2^< 0.84). Genotype and genotype x year interaction are highly significant factors across all three years in the multi-year ANOVA (**Table 4A**). Multi-year broad-sense heritability (H^2^) is moderate at 60 DAS (0.57< H^2^< 0.64) and high at 100 DAS. (0.88< H^2^< 0.89). Excluding 2017 data did not substantially change heritability estimates or which factors were considered significant (**Table 4B**). P-values for flowering-percentage ANOVA results are available in **Supplementary Tables 1**-**4**.

**Table 3A:** Single year ANOVA (2016-2018) of % flowering (60 DAS).

Source of Variation	Flowering % (60 DAS)								
	2016			2017			2018		
	df	F	p	df	F	p	df	F	p
Accession	678	9.575	***	694	7.295	***	680	13.519	***
Block	1	NS	NS	1	4.788	*	1	0.482	NS
Residuals	631			689			640		
H^2^	0.9			0.87			0.93		

Broad-sense heritability (H^2^) is very high for all three years studied. P-values in **Supplementary Table 1**.

Statistically significant at *p ≤ 0.05; ***p ≤ 0.001; NS, otherwise.

ANOVA results indicate that genotype is a highly significant factor for mid-season flowering habit.

**Table 3B:** Single year ANOVA (2016-2018) of % flowering (100 DAS).

Source of Variation	Flowering % (100 DAS)								
	2016			2017			2018		
	df	F	p	df	F	p	df	F	p
Accession	678	5.635	***	694	5.142	***	680	6.239	***
Block	1	7.315	***	1	24.493	***	1	0.64	NS
Residuals	631			689			640		
H^2^	0.83			0.81			0.84		

Broad-sense heritability (H^2^) is high for all years studied. P-values in **Supplementary Table 2**.

Statistically significant at ***p ≤ 0.001; NS, otherwise.

ANOVA results indicate that genotype is a highly significant factor for mid-season flowering habit in every year studied.

**Table 4A:** Multi-Year (2016-2018) ANOVA for flowering (%) (60 DAS) and bolting (%) (100 DAS) results indicate that genotype and genotype x year interaction are highly statistically significant factors in both traits across all three years.

Source of Variation	Flowering % 60 DAS			Flowering % 100 DAS		
	df	F	p	df	F	p
Accession	657	17.493	***	657	5.688	***
Year	2	5.753	**	2	8.572	***
Accession x Year	1310	4.821	***	1310	1.428	***
Block within Year	3	0.780	NS	3	9.627	***
Residuals	1889			1889		
H^2^	0.64			0.88		

Multi-year broad-sense heritability (H^2^) is moderately high for both traits. P-values in **Supplementary Table 3**.

Statistically significant at **p ≤ 0.01; ***p ≤ 0.001; NS, otherwise.

**Table 4B:** Multi-year ANOVA (2016 & 2018) for flowering (%) (60 DAS) and bolting (%) (100 DAS) results indicate that genotype and genotype x year interaction are highly statistically significant factors across all three years.

Source of Variation	Flowering % 60 DAS			Flowering % 100 DAS		
	df	F	p	df	F	p
Accession	657	12.637	***	657	5.340	***
Year	1	6.165	**	1	6.776	**
Accession x Year	643	4.659	***	643	1.119	*
Block within Year	2	0.785	NS	2	4.239	**
Residuals	1234			1234		
H^2^	0.57			0.89		

Multi-year broad-sense heritability (H^2^) is moderately high for both traits. P-values in **Supplementary Table 4**.

Statistically significant at *p ≤ 0.05; **p ≤ 0.01; ***p ≤ 0.001; NS, otherwise.

### Trait correlations

**Figure 6:**
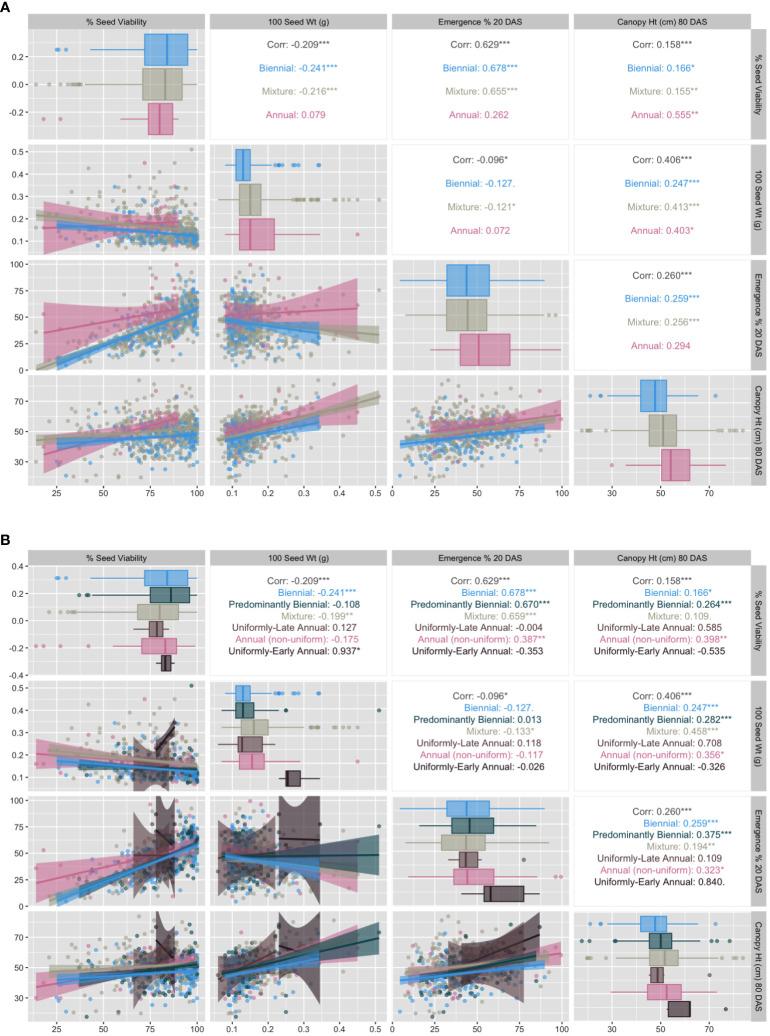
**(A)** Correlation matrix of vegetative growth traits factored by GRIN-Global flowering descriptors (biennial, annual, mixture) as defined by 100 DAS evaluation: 0% flowering (biennial), 100% flowering (annual), and 1% - 99% flowering (mixture) (2016 and 2018 marginal means). **(B)** Correlation matrix of vegetative growth traits factored by proposed CarrotOmics flowering habit trait ontology (2016 and 2018 marginal means). Pearson's correlations are statistically significant at *p ≤ 0.05; **p ≤ 0.01; ***p ≤ 0.001; NS, otherwise.

Using the strictest definitions for annual (100% flowering) and biennial (0% flowering), we explored vegetative trait correlations among the three traditional flowering habit categories (**Figure 6A**). Correlations among shoot-growth traits vary by flowering habit category. Correlation between seed viability and emergence was high for biennials (r = 0.68) and mixtures (r = 0.66) and very low and not significant for annuals (r = 0.26). Seed viability and seed weight had low negative correlation in biennials and mixtures, and was uncorrelated in annuals. Correlation between seed viability and late-season canopy height is moderate and positive for annuals (r = 0.56) and very low for biennials (r = 0.17) and mixtures (r = 0.16). Across all three flowering habits, emergence has low correlation with canopy height (80 DAS) and no correlation with seed weight. There also appeared to be a difference in mean and variation between biennial and annual accessions for canopy height. In the simple three-category model, mixtures tend to behave more similarly to biennials than to annuals, sharing very similar correlations among trait pairs, with one exception: the correlation between seed weight and canopy height was slightly higher for mixtures and annuals (r=0.41) than for biennials (r=0.25). There also appears to be a difference in mean and variation between biennial and annual accessions for canopy height.

We evaluated relationships among our trait ontology’s categories and subcategories (**Figure 6B**).

**Figure 7:**
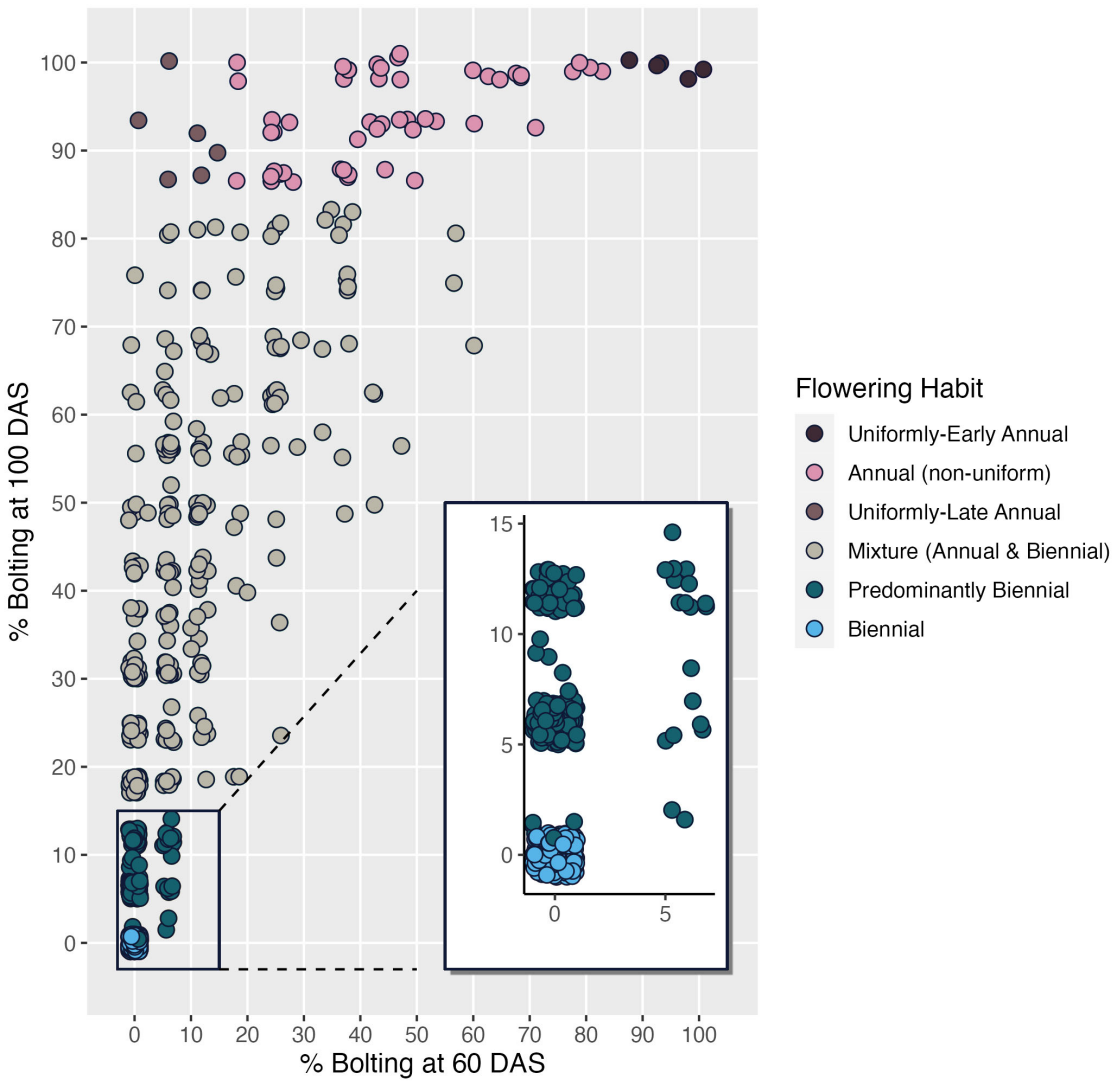
Scatterplot visualization of CarrotOmics flowering habit trait ontology based on estimated marginal means from 2016 and 2018.

Correlations among shoot-growth traits vary by flowering habit category. As with **Figure 6A**, correlation between seed viability and emergence is high for biennials (r = 0.68), predominantly biennials (r = 0.67) and mixtures (r = 0.66) and low to non-existent for annual populations (r = 0.0 for late annuals, =0.35 for early annuals and 0.39 for non-uniform annuals). Correlation between emergence and late-season canopy height is very high (r = 0.84) for uniformly-early annuals, as is the relationship between seed viability and seed weight (r = 0.94), and low for all other flowering categories. Data presented are on estimated marginal means across years. Uniformly-early annuals have the highest mean seed weight, emergence, and canopy height. We observed that 37% of accessions in this collection expressed some proportion of flowering by 60 DAS and that this increased to 70.5% by 100 DAS, suggesting that the 60 DAS measurement is insufficient to predict whether plants will flower, as only half of accessions that will flower by 100 DAS are flowering at 60 DAS (**Figure 7**). Many of the accessions that are flowering at 100 DAS may be late-flowering and could be of use to breeders, but would likely have been discarded using the prior classification system.

## Discussion

This study is the largest and most diverse global carrot germplasm collection yet evaluated for flowering at mid-season and end-of-season in multi-year trials. This diverse carrot collection demonstrates maximal breadth of variation for phenological flowering characteristics, with high broad-sense heritability estimates within and across years. This carrot flowering ontology attempts to capture this diverse range of flowering variation with additional categories based on perceived usefulness. High broad sense heritability for flowering reported in our study is similar to estimations in other carrot germplasm collections (Manikanta et al., 2018), and consistent with documented simple inheritance of flowering habit in carrot, with two recessive loci conditioning biennial habit (Alessandro & Galmarini, 2007; Alessandro et al., 2013; Wohlfeiler et al., 2019), and in other plants such as *Arabidopsis* (Michaels & Amasino, 2000), sugar beet (Abe et al., 1997), celery (Quiros et al., 1987), brassicas (Pelofske & Baggett, 1979; Baggett & Kean, 1989), and lettuce (Whitaker, 1944). Our study also agrees with Solberg and Yndgaard (2015), that the diversity within accessions for flowering habit is not well captured by the GRIN-global classification the genebanks’ information system, where accessions are categorized as biennial, annual, or mixture. The flowering habit assigned to accessions in this study largely disagreed with flowering designation in the GRIN-Global passport data, even when using the three original categories. Carrot accession flowering habit data in GRIN-Global was often collected on a limited number of carrot plants, and frequently on a single plant. This is further confounded by carrot’s outcrossing nature and that many accessions in this collection originated from landraces and open-pollinated varieties that were increased in the open field, giving way to the possibility of pollen contamination. Taken together, it cannot be assumed that the flowering habit of the individual plant will be reflected in the progeny. Additionally, flowering in carrot is mediated by a network of genes that are differentially influenced by photoperiod, temperature, and illumination intensity (Ou et al., 2017); consequently, the accession’s flowering phenotype at its collection origin could differ when trialed in other climatic conditions. This underscores the need for breeders to use more detailed phenotyping for germplasm they plan to introduce into their program in their respective target environments.

Descriptive statistics and ANOVA results are consistent: flowering percentage varied somewhat by year, with the starkest differences in 2017, a year with poor stand establishment that overestimated biennials and underestimated mixtures compared with 2016 and 2018, which had more comparable values for flowering-category counts (**Table 4A**). Our data suggests that a year with poor stand establishment, such as 2017, may overestimate the number of biennials. One possible reason for this in our trial is that uniformly-early annuals, with higher and perhaps earlier emergence, were damaged by hail, as well as early-emerging seedlings from mixed populations, but the later emerging seedlings survived. Mixtures accounted for 36% of the collection, and may contain some amount of early-flowering annual seedstock. Consequently, weather events that eliminate early-emerging plants may bias the resulting stand against annuals and in favor of biennials. Senescence of early annuals can also occur between 60 DAS and 100 DAS, which can in turn increase the perception of biennials. Though this happened infrequently (four of 695 accessions in our study), this can be detected and handled in the data when there is a *decline* in flowering percentage from 60 DAS to 100 DAS. Population parameter data for 2016 indicates that far more plants (37.7%) had signs of flowering at 60 DAS than in 2017 and 2018 (4.6% - 9.4%). This could have been due to early-season weather events that accelerated flowering in 2016.In large plant breeding trials, there is a need to balance efficiency and precision of measurements. Often large trials must sacrifice some precision for efficiency. Our five-point scoring system increases efficiency, with each plot taking fewer than five seconds to evaluate. High broad-sense heritability estimates for all flowering traits in all years (0.81< H^2^< 0.93) suggests that our methodology successfully detects genetic signals for flowering habit at both time points, at least in a diverse collection. Directly measuring flowering percentage on a continuous scale may result in more precise estimates, but gains may be trivial and unnecessary compared to the time spent on such a large collection. Extreme values in scatterplots of **Figure 6B** boxplots appeared to have little leverage or influence on slope. The three annual subcategories we created in **Figure 6B** enabled us to observe that the correlation we see in annuals in **Figure 6A** is driven by the uniformly-flowering early annuals. However, this correlation could be driven by genetic drift and small sample size (n=5). The inclusion of wild germplasm, which tends to flower early, in a future study could clarify this interpretation.

### Flowering trait ontology

A fundamental purpose of crop ontology is to create a descriptive and consistent vocabulary for crop traits, facilitating communication across collaborators and comparison of data between trial years, locations, and breeding programs (Shrestha et al., 2012; Walls et al., 2012). We have provided improved descriptions for flowering habit characteristics in carrot, including standard methodologies and time-frames for trait evaluation. Previous studies in carrot have used “early”/”late” as modifiers to flowering habit in the first growing season and second growing season, respectively. The term “annual” has conventionally been used synonymously with “early-flowering”, while the term “biennial” has been used synonymously with “late-flowering” (Alessandro & Galmarini, 2007; Alessandro et al., 2013; Wohlfeiler et al., 2019; Simon & Grzebelus, 2020; Wohlfeiler et al., 2021). However, confusion arises when early-flowering has also been used to describe wild carrot plants that flower earlier in the first growth season than cultivated annuals, which tend to flower later in the same season (Simon & Grzebelus, 2020). Furthermore, recent research suggests that a gradient of vernalization requirements exists within annuals and biennials (Wohlfeiler et al., 2021) – we propose a standardized vocabulary to refer to this germplasm.

While flowering has been typically measured at 100 DAS, the 60 DAS measurement enabled differentiation between annuals that flower uniformly early in the season (uniformly-early annuals) from annuals that flower uniformly at the end of the season (uniformly-late flowering annuals) (**Figure 5**). As posited by Wohlfeiler et al. (2019), these accessions could represent a range of vernalization requirements for flowering, with later flowering annuals needing more cold exposure and uniformly-early annuals needing far less, if any, cold exposure. Mixtures, which did not have a clear threshold defined previously, are entries with both biennial and annual plants in intermediate quantities, which we defined as between 1% and 85% flowering. We created a useful subcategory of mixture – predominantly biennial (n=160) – that constitutes a sizable 24% of accessions in this germplasm collection. Predominantly biennial accessions contain fewer than 15% flowering plants at the end of the season. Given the simple inheritance of biennial habit in carrot (Wohlfeiler et al., 2019), it is practical to select biennial plants and eliminate annual plants from a predominantly biennial population with other favorable traits for breeding. The establishment of the predominantly biennial subcategory nearly doubles the availability of germplasm with commercial potential, from 197 biennials to 357 biennials and predominantly biennials, accounting for 54% of the germplasm collection. This subcollection is a useful source of genetic diversity for breeders.

In this crop ontology, we propose categories that more fully describe the flowering habit of cultivated carrot, and we encourage carrot researchers to utilize and expand upon the descriptive terminology we provide in **Table 1** and **Figure 3**. In this ontology, “uniformly-early flowering” describes both precocity and uniformity of annual flowering, characterized by a high proportion of plants flowering at 60 DAS. “Late” describes only uniformity of end-of-season flowering, characterized by a low proportion of plants flowering at 60 DAS and a high proportion of plants flowering on harvest day or end-of-season. Following this logic, researchers can extend their evaluation of biennial carrot germplasm into the second season of growth. Collecting data on precocity of biennial flowering could identify uniformly-early biennials, which is important for carrot seed producers. This study provides a framework and an opportunity to study carrots in the second season of growth, and lays the groundwork for performing seed-to-seed phenotyping over the crop’s lifecycle.

Logically, the threshold of 85% for annual flowering means that these groups could also include mixtures. It would be more correct biologically to say that annuals are plants with 100% flowering at end of season, and any entries less than 100% flowering, and greater than 0% flowering, is a mixture. However, given that annuals are undesirable in temperate carrot production systems, we found little practical use in defining a “predominantly annual” population. It is more likely that non-flowering plants in an annual population at the end of the season are late-flowering annuals rather than biennials. Furthermore, breeding programs typically avoid intercrossing temperate (biennial) and subtropical (annual) carrots, and rarely use wild germplasm, given that substantial new challenges outstrip the benefits of such a cross (Simon & Grzebelus, 2020). The accessions identified, as well as the methodology provided for the identification of uniformly-late annuals, could prove useful for carrot breeders in subtropical climates or semi-arid climates, where uniformly-late annuals are essential for successful cultivar development; carrots adapted to subtropical regions often flower with reduced exposure to cold and tend to flower prolifically in temperate regions. In subtropical climates, carrots are managed as late-flowering annuals, with some plants used to produce the root crop and the remainder generate the seed stock in the same season. Biennial plants are unsuited to subtropical and semi-arid market, as they do not contribute to the seed crop.

The ability to distinguish between uniformly-early annuals and uniformly-late annuals is present at 60 DAS and lost by 100 DAS (**Figures 2**, **4**, **5**), illustrating that the additional subcategories are distinct from one another in all three years evaluated. This demonstrates the need to evaluate flowering at two timepoints to capture the phenological variation in this population. The 60 DAS measurement alone is too early to identify the true biennials, but crucial when combined with the 100 DAS measurement for distinguishing uniformly-early annuals from uniformly-late annuals. The 100 DAS measurement alone is too late to differentiate the uniformly-early annuals from uniformly-late annuals, but critical to differentiate uniformly-late annuals from biennials, as well as identifying predominantly biennial populations. Our 100 DAS flowering evaluation allows us to further distinguish between accessions with uniformly-early annual habit (0.75% of accessions), uniformly-late annuals (0.9% of accessions), and the remaining annuals with non-uniform flowering (7.5% of accessions).

Identification of predominantly biennial accessions provides improved characterization of mixed population accessions and nearly doubles the availability of germplasm with commercial potential (n=357) for temperate areas of root production, compared with strictly biennial germplasm (n=197). Custom core collections could be curated from this data for carrot breeders and researchers interested in new sources of genetic diversity for specific traits, climatic conditions, production uses, or market types (Brown, 1989) by filtering on data such as plant morphology, ecogeographical origin, molecular marker data, and genetic relatedness (Berger et al., 2013; Byrne et al., 2018; Corak et al., 2019; Corak, 2021). In the biennial and predominantly biennial custom core collection, a minicore can be curated from our shoot-growth phenotypic data for traits such as plant height, canopy coverage, emergence, or available data for any trait of interest. Cores that maintain diversity while also maximizing desirable traits have great utility to breeders. The upper threshold for emergence is just as high in the biennial and predominantly biennial groups, which demonstrates the advantage of evaluating this custom core collection more closely for agronomically important traits. Agromorphological data has been leveraged to create core collections in sweet potato (Huamán et al., 1999), potato (Huamán et al., 2000), groundnut (Upadhyaya et al., 2003), pigeonpea (Reddy et al., 2005), maize (Malosetti & Abadie, 2001; Li et al., 2005; Risliawati et al., 2023), safflower (Dwivedi et al., 2005), yam (Girma et al., 2018), walnut (Mahmoodi et al., 2019), pomegranate (Razi et al., 2021), lentil (Tripathi et al., 2022), and Indian mustard (Nanjundan et al., 2022). Corak compared methods for creating custom core collections in a subset of 433 accessions from our study’s carrot diversity panel, and found that custom methods combined with representative methods built cores balanced for genetic representation and enriched for desirable phenotypes, though it is important to note that carrot has low population structure (Corak et al., 2019; Corak, 2021). Similarly, the mixtures and annuals we identified in this collection can be used as sources of genetic diversity by carrot breeders and researchers targeting carrots for subtropical/semi-arid climates.

### Limitations of our study

There is a potential bias in every cultivated germplasm collection, as traits that are considered useful are relative to culture, production system, environment, technological access, local economy, and myriad unmeasurable factors. One bias inherent in the USDA cultivated carrot germplasm collection is preference for biennial germplasm, as that is what is grown in the U.S. Other gene banks may have higher diversity and larger samples for annual habits. We expect to see varying proportions of flowering in carrot germplasm, with the least in biennial cultivated carrot bred for cool temperate climates, and increasing amount of flowering in annual cultivated carrot bred for subtropical/semi-arid climates and wild carrot, indicating that the proportion of flowering in a population is relative to the germplasm under evaluation. Previous studies on U.S. commercial carrot cultivars, which have been selected for bolting resistance, had insignificant amounts of bolting, such that they were noted but not analyzed (Luby et al., 2016). In wild germplasm, most were bolting in early planting but all were non-bolting in later plantings (Solberg and Yndgaard, 2015), which could be due to cooler spring with sufficiently low temperatures to induce vernalization, and a warm, temperate summer resulting in no bolting. These varying results likely reflect the genotypic base of the carrot germplasm and the environment under study. Similarly, biennial genotypes in warm climates will never flower, while warm-acclimated annuals in heat-stressed environments may flower readily and prolifically (Simon et al., 2019). This study is limited to three years in one temperate environment. With this said, this environment is a commercially relevant region, with Wisconsin ranking in the top 3 U.S. states for carrot root crop production. Studies are underway to characterize this USDA germplasm collection in multiple other commercially relevant temperate carrot production environments. Accessions characterized in Wisconsin may not be stable in other growing regions. Climate warming could stimulate early-flowering or increased total flowering in germplasm we have already characterized.

### Conclusions and recommendations

Motivations for this flowering ontology were twofold: to attempt to understand the essential nature of carrot flowering phenology and to promote utilization of diverse germplasm by way of its characterization for agronomically critical traits. The former goal was satisfied by combining 60 DAS and end-of-season flowering data and identifying subtle but significant distinctions among annuals that open questions into carrot’s life history and domestication. The ontology provides trait definitions and methods for measuring flowering habit in diverse germplasm. Users of this ontology can set their own threshold based on what they see as tolerable for their own program. Future evaluations can be improved by overseeding accessions with low germination or low emergence to achieve the sample size required for accurate characterization. To better elucidate the relationship between trait stability in other temperate and economically relevant carrot production regions, genomic data and multi-environmental data will be integrated with this study’s phenotype data in future carrot diversity panel studies on QTL x E. Multi-environmental GWAS studies in temperate, subtropical, and semi-arid climates will facilitate molecular characterization of flowering habit in other *Daucus* germplasm collections.

We were also motivated by utility, which is the primary concern of breeders evaluating diverse germplasm. Identification of predominantly biennial germplasm fulfilled this goal, expanding the availability of genetic backgrounds that can be leveraged in temperate breeding programs. This evaluation has improved access to useful plant introductions in mixtures by identifying predominantly biennial accessions, doubling the size of the commercially promising accession gene pool for temperate carrot production regions. Given the relatively simple inheritance of biennial flowering habit, breeders for temperate root production can select biennial individuals out of predominantly biennial germplasm or other mixed flowering populations. However, selections should be evaluated for other agronomic traits and validated in multi-year, multi-environment trials in target locations. Data from canopy studies can be used in combination with flowering studies to identify accessions with high emergence and vigorous shoot growth for temperate climates. Similarly, the mixtures and annuals we identified can be leveraged as sources of genetic diversity by carrot breeders and researchers targeting carrots for subtropical and semi-arid climates. As such, flowering habit characterization has increased access to genetic resources with baseline commercial potential and provided useful data and methods to global users of carrot germplasm.

While within-accession diversity can be a challenge for *ex situ* conservation systems (Solberg and Yndgaard, 2015), we propose leveraging it as an opportunity to perform selection for desirable ecotypes, enabling identification of accessions with flowering traits required by local markets. We have provided a roadmap for evaluating and characterizing flowering habit in vegetable crops with mixed lifeforms, and this methodology is immediately useful to breeders and users of carrot PGR. Evaluating flowering habit as a gradient, rather than a binary trait, expands availability of commercially viable germplasm, further lowering the barrier to utilization of carrot PGR.

## Data availability statement

Data on this study's accessions are hosted on the CarrotOmics database. https://www.CarrotOmics.org/file/409952. Researchers may requests these accessions through the USDA Germplasm Resources Information Network (GRIN) database of the U.S. National Plant Germplasm System (NPGS). https//npgsweb.ars-grin.gov/gringlobal/search.

## Author contributions

JL: Conceptualization, Data curation, Formal analysis, Investigation, Methodology, Project administration, Software, Supervision, Validation, Visualization, Writing – original draft, Writing – review & editing. ML: Formal analysis, Software, Validation, Writing – review & editing. JD: Conceptualization, Data curation, Formal analysis, Funding acquisition, Methodology, Project administration, Resources, Software, Supervision, Validation, Writing – review & editing. PS: Conceptualization, Funding acquisition, Methodology, Project administration, Resources, Supervision, Validation, Writing – review & editing.

## Supplementary material

Supplementary Table 1 P-values of single-year ANOVA (2016-2018) for flowering (%) (60 DAS) results indicate that genotype is a highly significant factor in all three years studied. Statistically significant p-values in bold.

Supplementary Table 2 P-values of single-year ANOVA (2016-2018) of flowering (%) (100 DAS). Results indicate that genotype is a highly significant factor in every year studied. Statistically significant p-values in bold.

Supplementary Table 3 P-values of multi-year ANOVA for flowering (%) (60 DAS) and flowering (%) (100 DAS) results indicate that genotype and genotype x year interaction are highly statistically significant factors across all three years. Statistically significant p-values in bold.

Supplementary Table 4 P-values of multi-year ANOVA (2016 & 2018) for flowering (%) (60 DAS) and flowering (%) (100 DAS) indicate that genotype and genotype x year interaction are highly statistically significant factors across all three years. Statistically significant p-values in bold.
